# Cloning and Characterization of a Unique Cytotoxic Protein Parasporin-5 Produced by *Bacillus thuringiensis* A1100 Strain

**DOI:** 10.3390/toxins6061882

**Published:** 2014-06-18

**Authors:** Keisuke Ekino, Shiro Okumura, Tomoyuki Ishikawa, Sakae Kitada, Hiroyuki Saitoh, Tetsuyuki Akao, Takuji Oka, Yoshiyuki Nomura, Michio Ohba, Takashi Shin, Eiichi Mizuki

**Affiliations:** 1 Department of Applied Microbial Technology, Faculty of Biotechnology and Life Science, Sojo University, 4-22-1 Ikeda, Kumamoto 860-0082, Japan; E-Mails: oka@bio.sojo-u.ac.jp (T.O.); nomura@bio.sojo-u.ac.jp (Y.N.); shin@bio.sojo-u.ac.jp (T.S.); 2 Biotechnology and Food Research Institute, Fukuoka Industrial Technology Center, 1465-5 Aikawa-machi, Kurume, Fukuoka 839-0861, Japan; E-Mails: sokumura@fitc.pref.fukuoka.jp (S.O.); tishikawa@fitc.pref.fukuoka.jp (T.I.); saitou@fitc.pref.fukuoka.jp (H.S.); akao@fitc.pref.fukuoka.jp (T.A.); emizuki@fitc.pref.fukuoka.jp (E.M.); 3 Department of Bioscience and Bioinfomatics, Kyushu Institute of Technology, Iizuka, Fukuoka 820-8502, Japan; E-Mail: kitada@bio.kyutech.ac.jp; 4 Graduate School of Agriculture, Kyushu University, Fukuoka 812-8581, Japan; E-Mail: gunka@yk2.so-net.ne.jp

**Keywords:** *Bacillus thuringiensis*, parasporin, cry toxin, β-pore-forming toxin

## Abstract

Parasporin is the cytocidal protein present in the parasporal inclusion of the non-insecticidal *Bacillus thuringiensis* strains, which has no hemolytic activity but has cytocidal activities, preferentially killing cancer cells. In this study, we characterized a cytocidal protein that belongs to this category, which was designated parasporin-5 (PS5). PS5 was purified from *B. thuringiensis* serovar *tohokuensis* strain A1100 based on its cytocidal activity against human leukemic T cells (MOLT-4). The 50% effective concentration (EC_50_) of PS5 to MOLT-4 cells was approximately 0.075 μg/mL. PS5 was expressed as a 33.8-kDa inactive precursor protein and exhibited cytocidal activity only when degraded by protease at the C-terminal into smaller molecules of 29.8 kDa. Although PS5 showed no significant homology with other known parasporins, a Position Specific Iterative-Basic Local Alignment Search Tool (PSI-BLAST) search revealed that the protein showed slight homology to, not only some *B. thuringiensis* Cry toxins, but also to aerolysin-type β-pore-forming toxins (β-PFTs). The recombinant PS5 protein could be obtained as an active protein only when it was expressed in a precursor followed by processing with proteinase K. The cytotoxic activities of the protein against various mammalian cell lines were evaluated. PS5 showed strong cytocidal activity to seven of 18 mammalian cell lines tested, and low to no cytotoxicity to the others.

## 1. Introduction

*Bacillus thuringiensis* is a gram-positive, spore-forming bacterium that produces parasporal crystals during sporulation. Some of these crystal proteins, referred to as Cry proteins, show specific insecticidal activity. Thus, *B. thuringiensis* has been studied for its potential as a biological control agent, and has become one of the most effective and important microbial insecticides for the control of insect pests, including Lepidoptera, Diptera, and Coleoptera [1]. On the other hand, some of the other crystal proteins produced by *B. thuringiensis*, known as Cyt proteins, show broad cytocidal activity against invertebrate and vertebrate cells, including mammalian erythrocytes [2]. Furthermore, previous studies revealed that non-insecticidal *B. thuringiensis* strains are more common and more widely distributed in nature compared to insecticidal strains [3,4]. We previously reported a unique property of crystal proteins from non-insecticidal *B. thuringiensis* isolates, non-hemolytic but capable of preferentially killing cancer cells [5,6,7,8,9]. These characteristic crystal proteins are referred to as parasporins (PSs) [10]. Six PS families had been identified so far (PS1–PS6) by the Committee of Parasporin Classification and Nomenclature [11,12]. In this paper, we report extensive information of PS5. The sequence data of PS5 has already been analyzed and recognized as a PS family. PSs are categorized into two types based on their molecular mass. The larger molecular mass group includes PS1, PS3, and PS6, and the smaller one includes PS2 and PS4, as well as PS5. The larger-type PSs are expressed as precursor proteins with a molecular mass of *ca.* 80 kDa, and are processed to active forms of *ca*. 60 kDa. All three proteins of this type (PS1, PS3, and PS6) have a three-domain structure consisting of five-block sequences that are highly conserved across most Cry proteins. PS1 was the first PS to be characterized and is the most well studied PS protein. The predicted precursor molecular mass of PS1 is 81 kDa, which is processed to the active form consisting of a 15-kDa and 56-kDa heterodimer. The mechanism of PS1’s cytocidal action appears to be via apoptosis [9]. PS3, which is structurally similar to PS1, also has a typical three-domain structure with an 88-kDa precursor protein that is activated to form a 64-kDa mature protein by both N-terminal and C-terminal local proteolysis processing [13]. PS6 (described as a CP84 toxin) shares a similar structure with PS1 and PS3, and is processed from an 84-kDa precursor to form a mature protein of 73 kDa consisting of a 14-kDa and 59-kDa heterodimer [14].

On the other hand, the small-type PSs are *ca.* 30-kDa activated proteins that are proteolytically processed from precursors with a molecular mass of 33–37 kDa, and have a non-conserved three-domain structure. PS2 is the 30-kDa activated form processed from a 37-kDa precursor. It is structurally similar to aerolysin-type β-pore-forming toxins (PFTs) [15]. This strongly suggests that PS2 exerts its cytocidal activity via pore formation followed by cell lysis [16]. In fact, PS2 forms oligomers (>200 kDa) in the presence of membrane proteins, as has been observed in other β-PFTs [17]. PS4 has a predicted molecular mass of 27 kDa, which is proteolytically processed at the C-terminal region from a 30-kDa inert precursor [18]. Some data suggest that PS4 might cause pore formation in the cell membrane similar to PS2 [19]. PS5, reported in this study, is categorized into this small molecular PS type. 

## 2. Results and Discussion

### 2.1. Purification of a Cytotoxic Crystal Protein from B. thuringiensis Strain A1100

**Figure 1:**
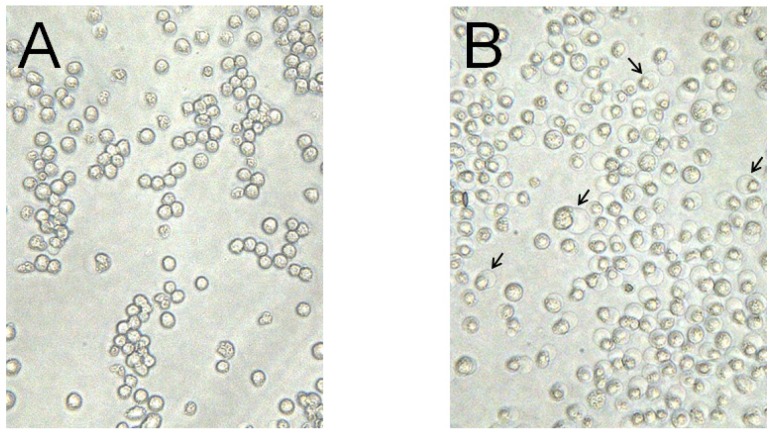
Cytopathic effect of crystal protein produced from *B. thuringiensis* A1100 on MOLT-4 cells. The parasporal inclusions were solubilized in alkali buffer and treated with proteinase K. (**A**) Mock-inoculated cells; (**B**) The cells were incubated with the solubilized protein at 37 °C for 1 h. Arrows indicate ballooned cells caused by administration of the cytotoxic protein.

**Figure 2:**
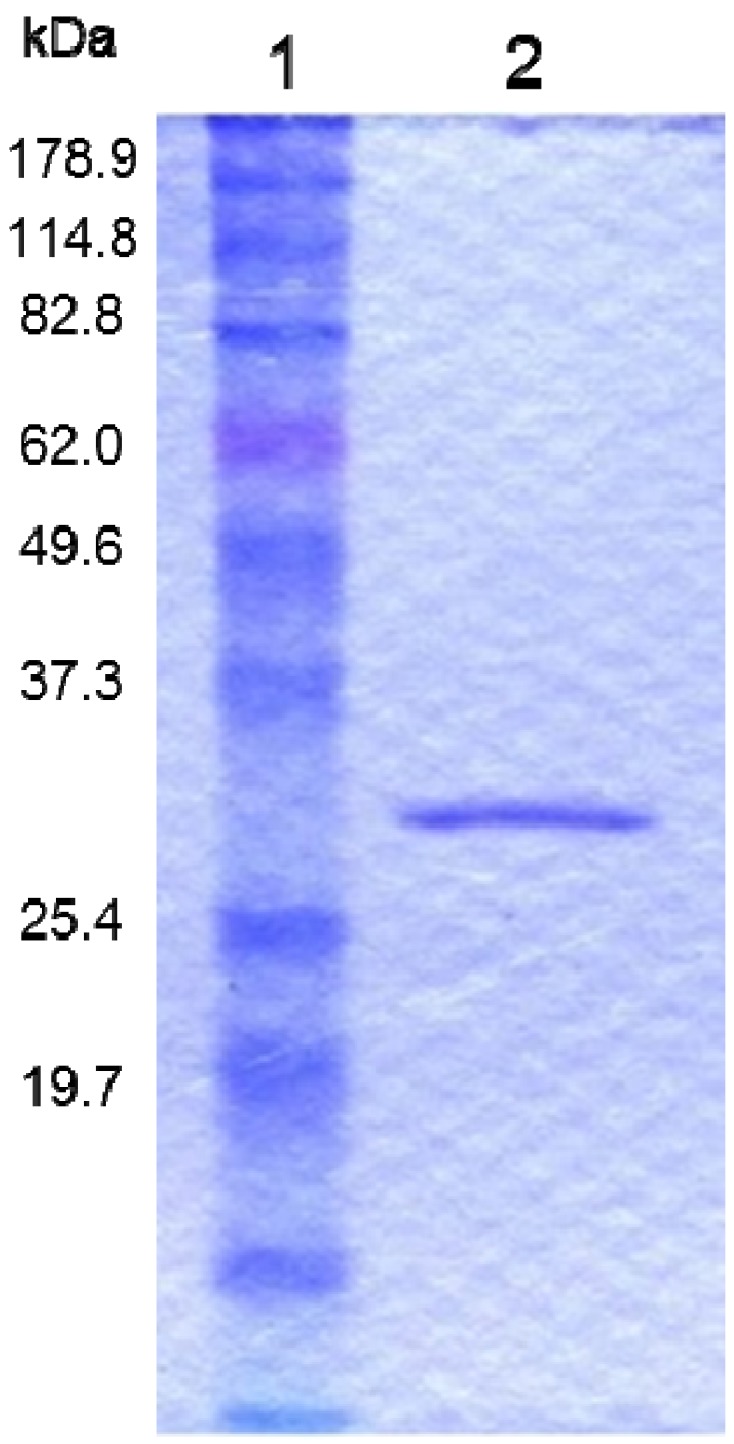
Protein profiles of the purified cytotoxic protein from *B. thuringiensis* A1100. Lane 1, molecular mass standards. Lane 2, purified 30-kDa protein.

**Figure 3:**
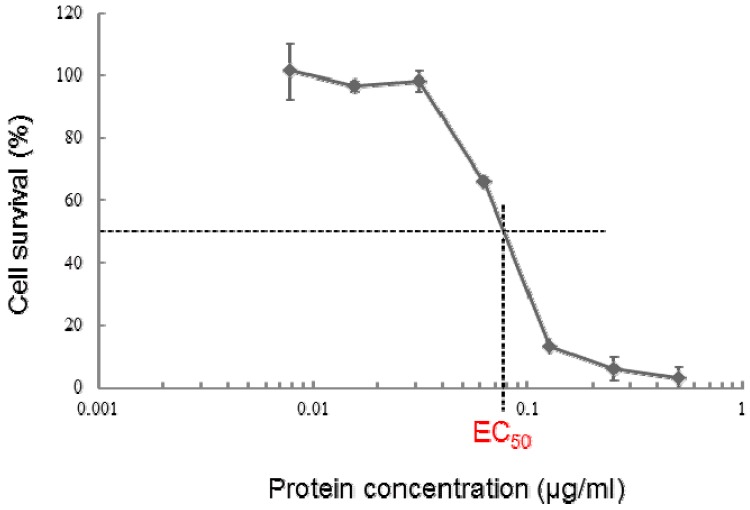
Dose-response curves for the purified 30-kDa protein from *B. thuringiensis* A1100 against MOLT-4 cells. The cell survival rate was evaluated using the MTT assay 20 h post-administration. The data are the means ± SDs of triplicate analyses.

Parasporal inclusions of the strain A1100, which were confirmed with a phase-contrast microscope, were only observed when incubated on the agar-plate culture for approximately one week. The parasporal inclusions were solubilized with alkali buffer and proteolytically processed with proteinase K for protein activation. The activated inclusion protein exhibited a strong cytopathic effect (CPE) on MOLT-4 cells within 1 h. The CPE could be monitored under a phase-contrast microscope. The characteristic change of a ballooning cell shape was observed when cells were treated with this toxic protein (Figure 1). In contrast, when the solubilized inclusions were not treated with proteinase K, no cytocidal activity was induced. In addition, no hemolytic activity against sheep erythrocytes could be detected (data not shown). Purification of the toxic protein was performed to monitor the CPE for MOLT-4 cells. Ammonium sulfate precipitation and several chromatographic steps were applied to purify this toxic protein (see Experimental Section). Density or sucrose gradient centrifugation is performed well, typical for the parasporal body purification in *B. thuringiensis*. However, this method cannot process a large quantity of samples. Thus, we carried out a chromatographic technique commonly used for protein purifications. The 30-kDa protein was purified to apparent near homogeneity and could be confirmed as one protein band in sodium dodecyl sulfate-polyacrylamide gel electrophoresis (SDS-PAGE; Figure 2). The dose-response curve of the purified protein to MOLT-4 cells was monitored by using a 3(4,5-dimethylthizol-2-yl)-2,5-diphenyltetrazolium bromide (MTT) assay, and the 50% effective concentration (EC_50_) value obtained 20 h after administration was approximately 0.075 μg/mL (Figure 3).

### 2.2. Genetic Characteristics of the Cytotoxic Protein

**Figure 4:**
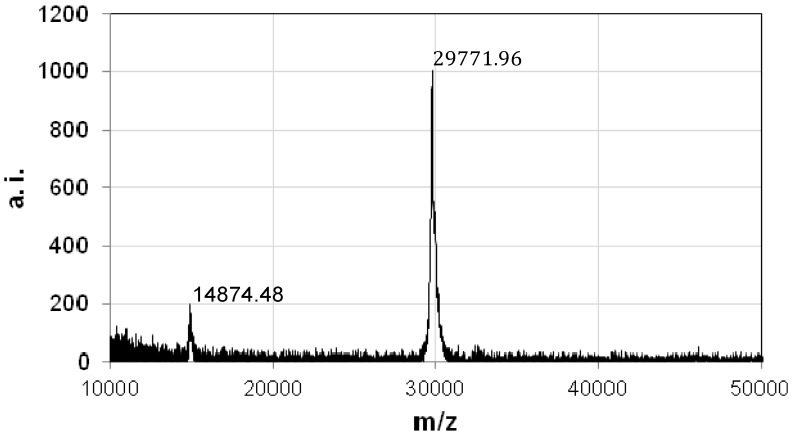
Molecular mass of the purified 30-kDa protein was determined by MALDI-TOF mass spectrometry. Arbitrary index a.i.; charge-to-mass ratio, m/z.

Sequence of the N-terminal region of the 30-kDa polypeptides and one of the internal fragments were determined by Edman degradation, and 12 and 10 amino acid residues were obtained, respectively. The primers were designed on the basis of the obtained polypeptides sequences. The 680-bp partial DNA fragment of the coding sequence could be amplified from the total DNA of strain A1100 by polymerase chain reaction (PCR) with these primers. This fragment was then used as a DNA probe for colony hybridization to isolate the entire coding gene. Several positive clones harboring the 3.4-kb *Pst*I fragment and the entire coding region were obtained. The cloned gene was 918 bp long, encoding a polypeptide of 305 amino acid residues with a predicted molecular weight of 33,797. According to the deduced amino acid sequence from the coding sequence, position 2–16 of the N-terminus (AIFDVEADLIDN) was identical to that of the purified 30-kDa protein. The internal sequence (AQALYTDRNE) of the 30-kDa protein was found in the deduced peptide sequence at position 214–223. The N-terminal amino acid sequence of the proteinase K-activated protein was identical to the N-terminus of the deduced coding sequence. Therefore, this cytotoxic protein was expressed as a 33.8-kDa non-toxic precursor and then processed to the active 30-kDa protein only when it was treated with proteinase K, which lost the C-terminal 3.8-kDa fragment. To elucidate the amino acid processing position, the molecular mass of the purified 30-kDa protein was determined by matrix-assisted laser desorption/ionization-time-of-flight (MALDI-TOF) mass spectrometry. The molecular mass was estimated at 29,772 (Figure 4), which indicated that the non-toxic proprotein was digested at the Gly271 position to activate the protein. 

The protein encoded by the gene was designated as Cry64Aa1 by the *B. thuringiensis* toxin nomenclature committee [20] and designated as PS5Aa1 by the Committee of Parasporin Classification and Nomenclature [12].

In genetic sequence analysis, although no typical promoter sequence was identified upstream of the PS5Aa1-coding sequence, another open reading frame (ORF) was identified in this region. The ORF, which was only incompletely cloned, exists in the upstream region of the *PS5Aa1* gene and seems to encode a protein that shows slight similarity with a putative chitin-binding domain protein from *Bacillus cereus*. Thus, we suggest that the PS5Aa1-encoding gene is transcribed in a polycistronic fashion. However, the function of the protein encoded upstream of the *PS5Aa1* gene is currently unknown. 

Based on the results of sequence analysis, this cytotoxic protein has no conserved five-block sequences found in most of Cry proteins. The amino acid sequence was compared with those of other known proteins based on a Basic Local Alignment Search Tool (BLAST) search [21]. No significant homology was observed between the PS5 protein sequence and those of known proteins, including *B. thuringiensis* Cry and Cyt proteins. However, Position Specific Iterative (PSI)-BLAST (threshold = 0.001) analysis [21] showed that PS5 has slight homology with other proteins, including Cry toxins from *B. thuringiensis* strains and β-PFTs produced in certain bacteria [22]. The Cry proteins were identified as follows: Cry15Aa (producing bacteria, *B. thuringiensis* serovar *thompsoni*; sequence identity: 29%) [23], Cry33Aa1 (*B. thuringiensis* serovar *dakota*, 34%) [24], CryET33 (GenBank Accession No. AF038048, *B. thuringiensis*, 24%), Cry51Aa1 (*B. thuringiensis* strain F14-1, 21%) [25], and Cry60Aa (*B. thuringiensis* serovar *jegathesan*, 28%) [26]. PS5 homology to β-PFTs was as follow: α-toxins from *Clostridium septicum* (sequence identity; 15%) [27], aerolysins from *Aeromonas hydrophila* (13%) [28], and ε-toxin from *Clostridium perfringens* (13%) [29]. The result of the PSI-BLAST analysis for PS5 was quite similar with that previously reported for PS4 [19]. In addition, PS5 shows 19% homology with PS4Aa1, which is also a kind of β-PFT [30]. 

### 2.3. Preparation of the Recombinant PS5 Protein

**Figure 5:**
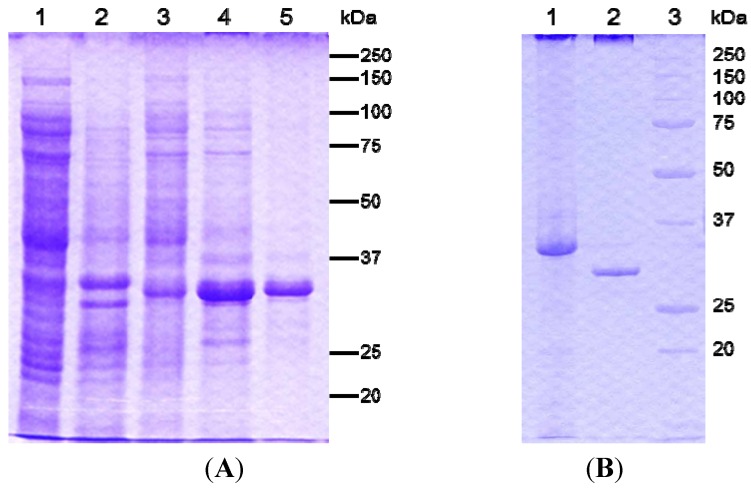
Analysis of the recombinant PS5 protein expressed by *E. coli* BL21 (DE3) harboring the plasmid pET-33k. (**A**) Lane 1, uninduced soluble fraction (control). Lane 2, uninduced insoluble fraction (control). Lane 3, IPTG-induced soluble fraction. Lane 4, IPTG-induced insoluble fraction. Lane 5, alkali-solubilized insoluble 33-kDa protein; (**B**) Lane 1, alkali-solubilized insoluble 33-kDa protein purified by a nickel-chelating column. Lane 2, the protein of lane 1 treated with proteinase K. Lane 3, molecular mass standards.

For the expression of two kinds of recombinant PS5 proteins, the genes encoding the full-length and C-terminal-processed active proteins were amplified by PCR and cloned into the pET32b vector at the *Nde*I and *Xho*I sites. The plasmid for the expression of full-length protein gene was confirmed with an in-frame His tag. These plasmids, pET-33k and pET-30k for the full-length and C-terminal eliminated fragment, respectively, were used to transform *Escherichia coli* BL21 (DE3) cells. Synthesis of each protein was confirmed after induction for 4 h with isopropyl β-d-1-thiogalactopyranoside (IPTG). Both proteins were synthesized as intracellular inclusion bodies in *E. coli*. The inclusions were solubilized and purified as described in the Experimental Section. The recombinant active-form protein expressed by pET-30k could be solubilized but had no cytotoxic effect on MOLT-4 cells. The solubilized recombinant full-length protein also had no cytocidal activity without protease digestion. Upon treatment with proteinase K, the 33-kDa protein was degraded into a 30-kDa protein and showed similar cytotoxicity to the native PS5 protein from strain A1100 (Figure 5). The activated recombinant 30-kDa protein also showed a rapid, strong cytotoxic effect on MOLT-4 cells as early as 1 h after administration. In addition, the EC_50_ to MOLT-4 cells of the purified recombinant 30-kDa protein was almost the same as that of the native toxic protein (data not shown). Thus, proteolytic processing was essential for activation of the cytotoxic protein, as is the case for most PSs. However, when the recombinant protein was expressed as a 30-kDa active form it had no cytocidal activity. This suggested that the C-terminal short fragments processed by proteinase in the precursor inhibited the protein’s activity and may be necessary for appropriate protein folding.

### 2.4. Cytotoxic Activity of PS5 against Various Mammalian Cells

The cytotoxic activities of the recombinant 30-kDa activated and purified proteins against various cultured mammalian cells were confirmed by conducting an MTT assay. The EC_50_ value for each cell line was determined (Table 1). As shown in Table 1, the sensitivity to the 30-kDa protein varied among the different cell lines tested. The recombinant protein was highly cytotoxic to MOLT-4, HepG2, TCS, HeLa, COS7, Vero, and Sawano cells, with all EC_50_ values lower than 0.1 μg/mL. For CACO-2, Jurkat, NIH3T3, CHO-K1, MRC-5, and UtSMC cells, only weak cytotoxicity with EC_50_ values from 0.1 to 1 μg/mL was observed. On the other hand, the 30-kDa active protein showed no cytotoxic effect on U937 and HC cells, with EC_50_ values greater than 10 μg/mL. Thus, there was no clear rule of cytotoxic specificity of this protein observed for the cultured mammalian cells, similar to other PSs.

With respect to the cytotoxic action mechanism of PSs, PS2, and PS4 were reported to act like β-PFTs, which cause increased permeability to susceptible cells. Both of these proteins formed SDS-resistant oligomers under the condition in which there is the plasma membrane of target cells, which is the commonly observed mechanism underlying the plasma membrane damage caused by β-PFTs. However, the actions of PS2 and PS4 mainly differ with respect to whether the process is cholesterol-dependent or -independent. In particular, PS2 requires cholesterol for its cytotoxic activity, similar to most PFTs [31], whereas the activity of PS4 is cholesterol-independent [19]. Similar to these PSs, PS5 is categorized into the small-size group of PSs, based on its molecular mass. In addition, PS5 shows the highest sequence similarity, although slight, to PS4 among all PSs. The results of the homology search using PSI-BLAST indicated that PS5 may also be included in the aerolysin-type β-PFT family along with PS4 [28,29,32]. Despite these similarities, considerable differences in cytotoxicity of mammalian cells were observed between PS5 and PS4, especially for Jurkat, HepG2, HeLa, COS7, NIH3T3, Vero, CHO, MRC-5, and UtSMC cells. Whereas PS4 showed no cytotoxic activity for any of the investigated normal cell lines [18], PS5 showed moderate cytotoxicity against MRC-5 and UtSMC cells. In addition, PS5 showed a broader cytotoxicity spectrum against the cultured cell lines. The mode of cytotoxic action of PS5 for these susceptible cells is currently unknown. Thus, it is necessary to further investigate these mechanisms by studying the effects of PS5 on plasma membrane damage and oligomer formation, as well as to identify the specific toxin receptor.

**Table 1:** Cytotoxic activity of recombinant parasporin-5 (PS5) protein against various cultured mammalian cells.

Cell line	Origin	EC_50_ (μg/mL)
MOLT-4	leukemic T cell, human	0.075
CACO-2	colon cancer, human	0.30
Jurkat	leukemic T cell, human	0.124
A549	lung cancer, human	4.41
HepG2	hepatocyte cancer, human	0.049
TCS	uterus cervix cancer, human	0.046
HL60	promyelocytic leukemia cell, human	1.079
K562	myelogenous leukemia cell, human	4.249
U937	DE-4 lymphoma cell, human	>10
HeLa	uterus cervix cancer, human	0.080
COS7	kidney cell, monkey	0.045
NIH3T3	embryo cell, NIH Swiss mouse	0.321
Vero	kidney cell, monkey	0.050
CHO-K1	ovary cell, chinese hamster	0.571
Sawano	uterus cancer, human	0.065
MRC-5	normal embryonic lung fibroblast, human	0.273
UtSMC	normal uterus, human	0.223
HC	normal hepatocyte, human	>10

## 3. Experimental Section

### 3.1. Bacterial Strains, Culture Media, and Preparation of Parasporal Inclusion Proteins

*B. thuringiensis* A1100, belonging to serovar *tohokuensis*, was grown on nutrient medium (0.1% meat extract, 0.1% polypeptone, 0.2% NaCl, and 2% agar, pH 7.6) at 30 °C for one week. After incubation, naturally lysed spores and parasporal bodies were collected and washed with deionized water three times. Then, the washed preparation was solubilized under alkali conditions with 50 mM sodium carbonate buffer (pH 10.0) containing 10 mM dithiothreitol (DTT) at 37 °C for approximately 1 h. After removal of insoluble materials (cellular debris and spores) by centrifugation at 15,000 × g for 10 min at 4 °C, the supernatant was used for the purification of cytocidal protein in parasporal inclusions.

### 3.2. Purification of Toxin Protein from Parasporal Inclusions

The solubilized parasporal inclusions were treated with proteinase K (final concentration, 10 μg/mL) at 37 °C for 90 min in order to activate the precursor protein, and then phenylmethylsulfonyl fluoride (final concentration, 1 mM) was added to stop the proteolysis. The protease-treated proteins were precipitated by ammonium sulfate saturation (up to 80%). The saturated solution was then centrifuged at 13,000 × g for 20 min at 4 °C and the pellet was suspended in 1/10 sample volume of a 20 mM sodium carbonate buffer (pH 10.0). The pellets and supernatants were checked for cytocidal activity against MOLT-4. The resuspended solution was applied to a column of Toyopeal Butyl-650M (Tosoh, Japan) equilibrated with 20 mM sodium carbonate buffer (pH 10.0) containing 1 M ammonium sulfate. The cytocidal fractions were eluted with a linear gradient (1–0 M) of ammonium sulfate. Active fractions were combined and dialyzed against 10 mM phosphate buffer (pH 8.0). The cytocidal active solution was applied to a column of hydroxyapatite HT (Bio-Rad Laboratories; Tokyo, Japan), which was previously equilibrated with 10 mM phosphate buffer (pH 8.0). The active fractions were eluted with a linear gradient (10–500 mM) of phosphate buffer (pH 8.0). Then, combined active fractions were concentrated and dialyzed against 20 mM Tris-HCl (pH 8.5) using an Amicon ultrafiltration cell, model 8050 (Amicon; Millipore; Bedford, MA, USA), comprising a 10,000-Da nominal molecular mass separation membrane (Ultracel 10-kDa regenerated cellulose; Amicon; Millipore). The cytocidal solution was applied to a column of TSKgel BioAssist Q (Tosoh, Japan) equilibrated with 20 mM Tris-HCl (pH 8.5). The active fractions were eluted with a linear gradient (0–0.5 M) of NaCl in 20 mM Tris-HCl (pH 8.5). The cytocidal fractions were pooled as the final product.

### 3.3. Mammalian Cells and Cell Culture

The cultured mammalian cell lines and culture conditions used in this study were almost identical to those described in Okumura *et al*. [18]. Cytocidal activities were examined for 18 cell lines in this study, whereas 20 cell lines were tested in the previous study.

### 3.4. Cytotoxicity Assay

MOLT-4 cells were cultured in RPMI1640 medium (Wako Pure Chemical; Osaka, Japan) containing 100 units/mL penicillin, 0.1 mg/mL streptomycin, and 10% fetal bovine serum at 37 °C in 5% CO_2_. MOLT-4 cells were routinely maintained for the investigation of cytotoxicity. Cytotoxicity assays were carried out in 96-well microtest plates. Ninety microliters of cell suspension including 2 × 10^4^ cells was added to each well in the plate and incubated at 37 °C for 1 d. Subsequently, 10 μL of the protein solution per well was used to investigate the cytocidal activity. The CPE was monitored under a phase-contrast microscope 1 h post-administration. The levels of cytotoxicity of the cytotoxic proteins were also evaluated by the MTT assay using a CellTiter 96 AQueous One Solution Cell Proliferation Assay Kit (Promega, Madison, WI, USA). The cell survival rate was determined by comparing the absorbance value with that of the control (without activated proteins, 100% viability) or 0.1% Triton X-100 treatment (0% viability). Experiments were performed in triplicate. The EC_50_ values of PS5 for each cell line were determined as described previously [18].

### 3.5. SDS-PAGE and Protein Determination

SDS-PAGE was performed following the method described by Laemmli [33]. After electrophoresis, the gels were stained with 0.1% Coomassie Brilliant Blue R-250 (Wako Pure Chemical; Osaka, Japan). The molecular masses of proteins were estimated by using molecular mass standards (Benchmark protein markers; GIBCO BRL; Carlsbad, CA, USA, and Precision Plus Protein Standard; Bio-Rad Laboratories; Hercules, CA, USA). Protein concentration was determined following the Bradford method [34] using a Bio-Rad Protein Assay Kit II (Bio-Rad Laboratories; Hercules, CA, USA). 

### 3.6. Mass Spectrometric Analysis

The molecular mass of purified PS5 from *B. thuringiensis* A1100 was determined on a MALDI-TOF mass spectrometer (Autoflex; Buruker Daltonics Inc.; Billerica, MA, USA). The details of the sample preparation, matrix, and molecular mass standard used for calibration are described previously [18]. 

### 3.7. N-Terminal and Internal Amino Acid Sequencing

To obtain partial amino acid sequences, the purified PS5 protein was reduced, S-alkylated, and digested with sequencing-grade TPCK-trypsin (Roche Molecular Biochemicals; Indianapolis, IN, USA) to achieve a protein/enzyme ratio of 50:1 in 50 mM NH_4_HCO_3_ (pH 8.0), and incubated for 16 h at 37 °C. The resulting peptides were separated by reversed-phase high-performance liquid chromatography (Wakosil-II 5C18AR column 4.6 mm× 250 mm; Wako Pure Chemical; Osaka, Japan) at a flow rate of 1 mL/min and monitored at 210 nm, eluting in 0.05% trifluoroacetic acid with a 0%–80% acetonitrile gradient using a TOSOH 8020 system (Tosoh). The N-terminal amino acid sequence of the PS5 protein and sequences of separated peptides of selected fractions were determined using a PPSQ-31A protein sequencer (SHIMADZU; Kyoto, Japan).

### 3.8. DNA Manipulation and PCR Experiments

Total DNA from *B. thuringiensis* strain A1100 was extracted using the CTAB method [35]. Plasmid DNAs of *E. coli* were isolated with a Labopass mini plasmid DNA purification kit (Hokkaido System Science; Sapporo, Japan). The oligonucleotides were purchased from Operon Biotechnology (Tokyo, Japan). PCR amplification was performed using KOD Plus DNA polymerase, as recommended by the manufacturer (Toyobo; Osaka, Japan), on a programmable thermal cycler (PC-701; Astec; Fukuoka, Japan). Restriction enzymes were used for gene manipulation as recommended by the manufacturer (Nippon Gene; Toyama, Japan). For Southern hybridization and colony hybridization, Hybond N+ membranes (Amersham Pharmacia Biotech; Uppsala, Sweden) were used. The DNA probes were labeled with digoxigenin (DIG) using the DIG Labeling and Detection Kit (Roche Diagnostics; Mannheim, Germany) according to the manufacturer instruction manual.

### 3.9. Gene Cloning and Sequencing

For the amplification of *PS5* internal sequences by PCR, two oligonucleotides were designed on the basis of the N-terminal amino acid sequence of the protein and one of the peptides obtained from TPCK-trypsin digests. The sequence of the former was AIFDVEADLIDN, corresponding to the DNA sense strand 5′-GCNATHTTYGAYGTNGARGCNGAYYTNATHGA-3′, while the latter sequence was AQALYTDRNE, corresponding to the antisense strand 5′-TTRTCNGTRTARTANARNGCYTGNGC-3′. To determine the sizes of restriction fragments containing the coding gene for the PS5 protein in Southern hybridization, the PCR products were labeled with DIG as the probe and used in DNA hybridization experiments with total DNA of strain A1100 digested with various restriction enzymes. The probe hybridized to a 3.5-kb *Pst*I fragment. Positive signals were visualized according to the instruction manual of the DIG Labeling and Detection Kit. Approximately 3–4-kb *Pst*I-digested fragments were excised from the agarose gel and purified using the Gene Clean kit II (Funakoshi; Tokyo, Japan). The DNA fragments were ligated into *Pst*I-digested pHSG396 plasmid (Takara Bio; Kyoto, Japan). The ligation mixture was used to transform *E. coli* XL1-Blue. Selection of the target sequence was carried out by colony hybridization with the PCR probe. DNA sequencing was performed by Operon Biotechnology.

### 3.10. Plasmid Construction for Expression of the Recombinant Protein

The coding region of the *PS5* gene was amplified from the plasmid containing the cloned *PS5* gene by PCR. The forward primer (5′-GGATCCGCATATGGCGATTTTTGATGTTGA-3′) contained an *Nde*I site (underlined) at the start codon of the gene. The reverse primer (5′-ACTCGAGTCTTTGTAAATACCTCTGATATT-3′) had a *Xho*I site (underlined) followed by DNA complimentary to the 3′-end of the gene. For synthesis of the active form of PS5, the gene of an appropriate region (from Met1 to Gly271) was amplified in a similar manner. The same forward primer indicated above was used for this amplification, whereas the reverse primer was 5′-ACTCGAG
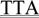
ACCTGGTAAAGGCGATT-3′ (underlined bases indicate the *Xho*I site, and the double-underlined bases indicate the TAA stop codon). The PCR products were ligated into the expression vector pET32b between the *Nde*I and *Xho*I sites. The plasmids were designated as pET-33k and pET-30k, respectively. *E. coli* XL1-Blue cells were transformed with the plasmids and plated onto Luria-Bertani (LB) agar containing ampicillin (100 μg/mL). 

### 3.11. Expression and Purification of Recombinant Toxin Proteins

*E. coli* BL21 (DE3) cells were transformed with the plasmids obtained from the XL1-Blue strains and were cultured in LB medium. Gene expression was induced with 0.1 mM IPTG at 37 °C for approximately 4 h. After harvesting the cells by centrifugation at 8000 × g for 1 min, the cells were suspended in 10 mM Tris-HCl (pH 7.5) and then lysed by sonication (Ultrasonic Disruptor UD-201; Tomy; Tokyo, Japan). The disrupted cell suspension was centrifuged at 15,000 × g for 10 min and the supernatant containing the soluble protein was removed. The pellet was solubilized with 50 mM sodium carbonate buffer (pH 10.0) at 37 °C for 90 min in the presence of 10 mM DTT. The soluble extract was recovered by centrifugation for 10 min at 15,000 × g. In the case of the full-length toxin protein expressed by pET-33k, the extract was loaded on a nickel-chelating Sepharose column (Amersham Pharmacia; His-trap) and eluted with 500 mM imidazole. The eluted fraction was then dialyzed overnight at 4 °C against 50 mM sodium carbonate buffer (pH 10.0) and then treated with proteinase K (final concentration, 10 μg/mL) at 37 °C for 1 h for activation. Then, the processed toxin protein was further purified using a Q Sepharose Fast Flow column (Amersham Pharmacia Biotech) equilibrated with 50 mM sodium carbonate buffer (pH 10.0) and eluted by a NaCl gradient (0–0.5 M). The protein’s purity was confirmed using SDS-PAGE.

### 3.12. Nucleotide Sequence Accession Number

The nucleotide sequence obtained in this study has been deposited in the GenBank database (accession number: AB555650.1). 

## 4. Conclusions

In this study, we isolated a 30-kDa protein from parasporal inclusions of *B. thuringiensis* serovar *tohokuensis* strain A1100 that shows cytocidal activity to leukemic cells. This protein shares little amino acid sequence homology with any other proteins, including Cry and Cyt proteins from *B. thuringiensis*. Thus, it constitutes a new class of Cry protein, designated as Cry64Aa1 and parasporin-5Aa1 (PS5Aa1). 
